# Determinants of malaria prevalence among children under 5 years of age in 17 sub-Saharan African countries: insights from Malaria Indicator Surveys

**DOI:** 10.1080/16549716.2026.2677281

**Published:** 2026-05-22

**Authors:** Alemneh Tadesse Kassie, Astewil Moges Bazezew, Tadesse Tarik Tamir, Berhan Tekeba, Gebreeyesus Abera Zeleke, Enyew Getaneh Mekonen, Helen Lamesgin Endalew, Mulugeta Wassie, Alebachew Ferede Zegeye

**Affiliations:** a Department of Clinical Midwifery, School of Midwifery, College of Medicine and Health Sciences, University of Gondar, Gondar, Ethiopia; b Department of Surgical Nursing, School of Nursing, College of Medicine and Health Sciences, University of Gondar, Gondar, Ethiopia; c Department of Pediatrics and Child Health Nursing, School of Nursing, College of Medicine and Health Sciences, University of Gondar, Gondar, Ethiopia; d Department of Medical Nursing, School of Nursing, College of Medicine and Health Sciences, University of Gondar, Gondar, Ethiopia

**Keywords:** Plasmodium infection, burden, under-fives children, household malaria survey, tropical African countries

## Abstract

**Background:**

Malaria remains a major public health burden among children under five in several Sub-Saharan African (SSA) countries, largely attributable to their underdeveloped immune systems, constrained access to healthcare services, and environmental conditions conducive to transmission.

**Objective:**

This study aims to identify the key determinants of malaria infection among children under five by examining both individual-level characteristics and community-level drivers within this vulnerable population.

**Methods:**

We conducted a secondary analysis of the Malaria Indicator Surveys (2016–2022), including 51,457 children under five from 17 SSA countries. A multilevel mixed-effects logistic regression model was applied, with significance set at *p* < 0.05. Determinants were reported as adjusted odds ratios (AORs) with 95% confidence intervals (CIs).

**Results:**

The analysis showed that more than one-third of children under five in several sub-Saharan African (SSA) countries were infected with malaria. At the individual level, significant risk factors included age (1–2 years: AOR = 1.46) and low household wealth index (AOR = 1.94). Protective factors were maternal primary education (AOR = 0.84) and the use of insecticide-treated bed nets (AOR = 0.80). At the community level, increased malaria risk was associated with rural residence (AOR = 2.69) and high community illiteracy rates (AOR = 1.76).

**Conclusions:**

The study findings highlight the sustained health threat that malaria poses to children under 5 years of age in many sub-Saharan African countries. Policymakers should prioritize reducing infections in vulnerable groups, expanding access to insecticide-treated bed nets, and promoting targeted health education to strengthen family and community awareness.

## Paper Context

- **Main findings:** Malaria affects over one-third of children under five in sub-Saharan Africa, shaped by both individual (age, household wealth) and community-level (rural residence, illiteracy) determinants.
- **Added knowledge:** The study demonstrates how socio-economic and educational disparities directly influence malaria prevalence among young children.
- **Global health impact for policy and action:** Targeted interventions expanding bed net coverage, strengthening health education, and addressing community-level inequities are essential to reduce malaria infections in vulnerable populations.

## Background

Malaria is a severe and potentially fatal disease caused by protozoan parasites of the genus Plasmodium, transmitted to humans through the bite of infected female Anopheles mosquitoes [1,2]. Children under five are especially susceptible to malaria because their immune systems are still developing and lack the full capacity to mount effective responses against infection [3,4]. In its 2024 World Malaria Report, the WHO highlights that the global malaria burden remains heavily concentrated in Africa, accounting for 95% of the estimated 263 million cases and related deaths worldwide. Children under 5 years represent 75% of these fatalities [5]. Globally, malaria cases reached an estimated 282 million in 2024, resulting in more than 600,000 deaths. Although current interventions including insecticide-treated nets, antimalarial medications, and vaccines are effective, they remain insufficient to fully address the challenges posed by weak health systems and limited resilience to external shocks such as political instability, economic crises, and climate variability [6,7]. Efforts to combat malaria are hindered by multiple challenges, including the parasite’s increasing resistance to antimalarial drugs, the mosquito vector’s growing resistance to insecticides, and persistent socio-economic barriers that limit access to preventive measures and healthcare services [8,9].

Malaria infection in sub-Saharan Africa (SSA) is shaped by a complex interplay of geographic, environmental, socio-economic, and demographic factors [10,11]. Lowland areas, with climatic conditions favorable for mosquito breeding, experience substantially higher malaria transmission rates compared to highland regions [12]. A recent study reported that children residing in lowland areas are approximately 15 times more likely to contract malaria than those living in highland regions, underscoring the critical influence of geography on malaria epidemiology [13,14].

Malaria remains a major health threat to children under five, driven by individual factors that heighten susceptibility. Age is a particularly critical determinant, with children between 6 months and 2 years experiencing the highest infection rates due to the absence of acquired immunity from prior exposure [15,16]. Gender also appears to play a noteworthy role, with studies suggesting that girls may have lower odds of contracting malaria compared to boys. The underlying reasons for this disparity are complex and remain insufficiently understood, warranting further investigation [17]. Maternal education is a critical determinant of malaria prevalence. Children of educated mothers generally experience better health outcomes, including a reduced incidence of malaria. Educated mothers are more likely to adopt preventive measures, such as consistent use of insecticide-treated bed nets, and to seek timely medical care when symptoms arise [18,19]. Socio-economic status and household conditions can exacerbate the malaria burden in vulnerable populations. Children from poorer households face higher odds of infection due to limited access to healthcare and essential preventive resources [20,21]. To sustain malaria programs, African governments must increase domestic funding and cultivate strong public – private partnerships, particularly in the context of funding constraints and emerging challenges such as climate change [7]. Evidence also underscores the protective influence of household environmental factors on malaria prevalence. The consistent use of bed nets, application of indoor residual spraying, and improvements in housing quality – such as durable flooring and roofing materials – significantly reduce the risk of malaria infection among children [22,23].

At the community level, socio-educational factors play a decisive role in malaria control. The 2025 WHO World Malaria Report highlights that communities with high illiteracy rates face greater malaria transmission risks due to limited awareness of prevention and treatment, whereas improved education and media exposure are associated with better uptake of insecticide-treated nets, chemoprevention, and timely treatment-seeking [24]. Conversely, active media use has been linked with improved knowledge and attitudes toward malaria prevention, underscoring the necessity of health education campaigns in reducing malaria transmission [25].

**Figure 1.**
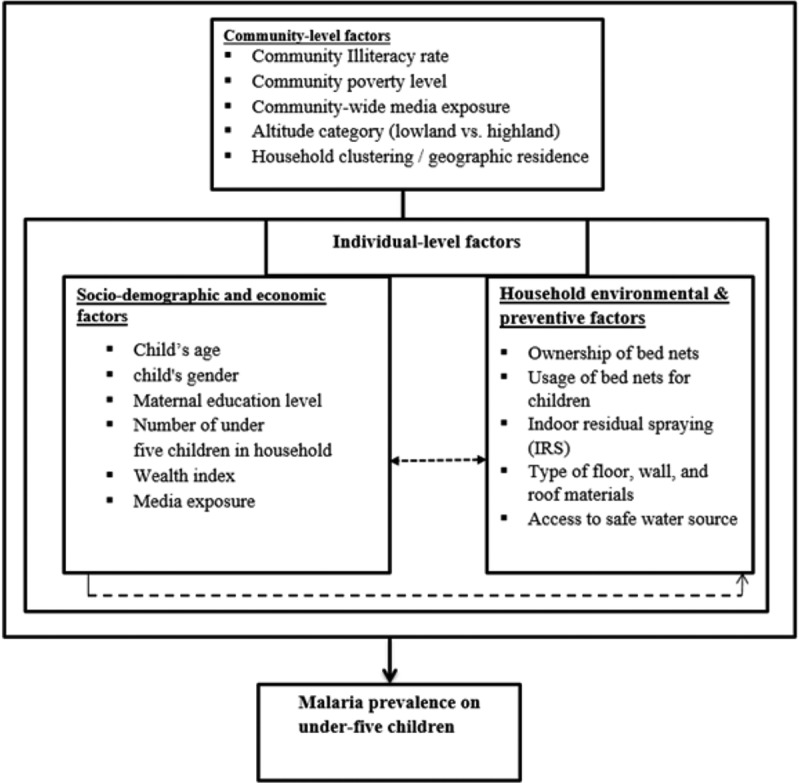
Conceptual framework for malaria prevalence among children under 5 years of age years.

Malaria infection in SSA is shaped by both individual and community-level factors. This study examines socio-demographic characteristics, household environmental conditions, and community influences (Figure 1) to provide a comprehensive understanding of infection determinants in children under five. Framed within the health and social context, the findings are expected to guide targeted interventions that reduce transmission and improve child health outcomes across 17 countries.

## Conceptual framework

### Method

Malaria prevalence among children under five was modeled as a binary outcome (infected/not infected) using multilevel logistic regression, incorporating both individual- and community-level predictors. Univariable analyses assessed crude associations, followed by multivariable models adjusting for confounders, with results expressed as adjusted odds ratios and 95% confidence intervals and with statistical significance set at *p* < 0.05.

Data were drawn from the Malaria Indicator Surveys (2016–2022) conducted in 17 sub-Saharan African countries: Burkina Faso, Cameroon, Ghana, Guinea, Kenya, Liberia, Madagascar, Mali, Malawi, Mozambique, Nigeria, Niger, Sierra Leone, Senegal, Togo, Tanzania, and Uganda. These nationally representative surveys targeted households with at least one child under five and assessed malaria positivity using rapid diagnostic tests. The datasets provide comprehensive information on household and community characteristics, enabling rigorous analysis of malaria determinants and supporting evidence-based public health initiatives.

This methodological framework enables a rigorous examination of how individual- and community-level determinants jointly shape malaria risk among children under five across diverse settings. Data were obtained from the DHS Program’s Malaria Indicator Surveys (MIS), ensuring the integrity and reliability of health and population trends. Datasets were selected based on the availability of malaria positivity data in under-fives, assessed using rapid diagnostic tests. Statistical analysis was conducted in Stata using multilevel mixed-effects logistic regression to account for the hierarchical structure of children nested within communities.

This secondary analysis provides a comprehensive understanding of factors contributing to malaria infection and their relationship to child morbidity and mortality. Findings are contextualized within broader public health initiatives, offering evidence to inform targeted interventions that can strengthen prevention and treatment strategies. Data are publicly available at The DHS Program – MIS Data.

Data collection was carried out by in-country research teams and statistical agencies, with technical assistance provided throughout the process. The surveys followed a two-stage sampling design, where enumeration units or ‘clusters’ were first selected from larger regional units within each country, and households were then randomly sampled within these clusters. The national representativeness of the surveys enhances the generalizability of the findings, while established statistical techniques reinforce the reliability of the results. The study defined children under 5 years of age malaria prevalence as its outcome variable, categorizing children who had been tested positive and those who had been tested negative for malaria as determined by healthcare providers during their screening visits.

## Study setting

**Figure 2.**
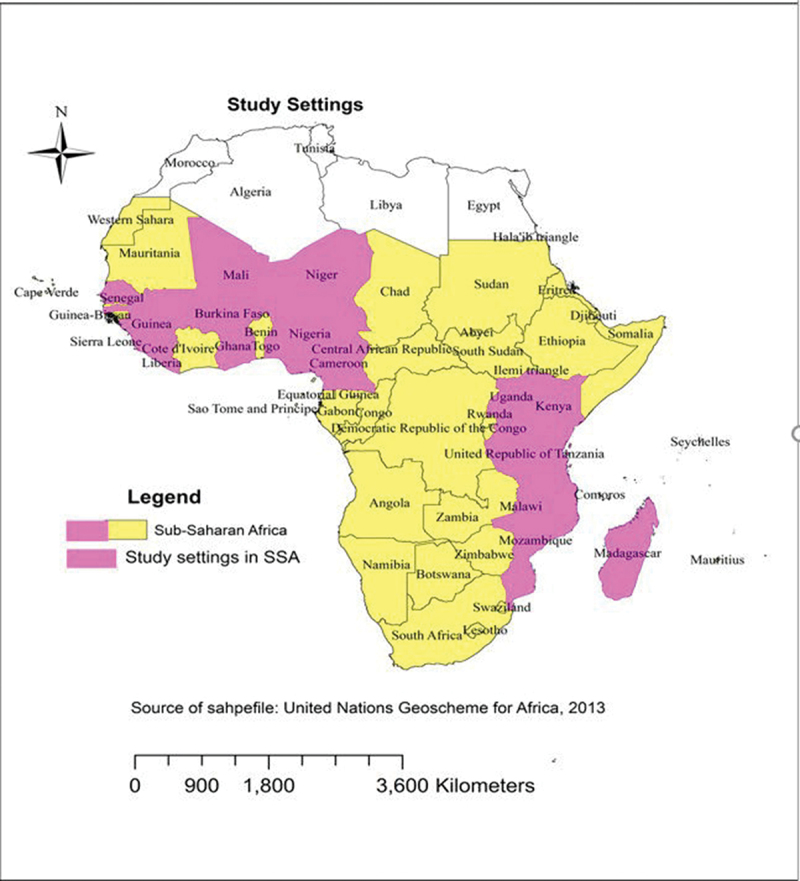
Map illustrating the study areas for malaria research among children under 5 years of age in Sub-Saharan Africa (SSA).

Sub-Saharan Africa (SSA), located south of the Sahara Desert, is divided into four regions: East, Central, West, and Southern Africa. Covering approximately 9.4 million square miles, it is home to about 1.3 billion people. Data for this study were drawn from Malaria Indicator Surveys (MIS) conducted between 2016 and 2022 in 17 countries: Burkina Faso, Cameroon, Ghana, Guinea, Kenya, Liberia, Madagascar, Mali, Malawi, Mozambique, Nigeria, Niger, Sierra Leone, Senegal, Togo, Tanzania, and Uganda. As surveys in other countries have not been conducted since 2016, only updated sources from the past 7 years were included. Using ArcGIS, malaria infections among children under five were visually represented, and the accompanying map highlights the specific areas from which data were collected (Figure 2).

## Study design and period

This study utilized a multilevel, community-based, cross-sectional design with mixed effects to evaluate malaria infection among children under 5 years of age in the 17 countries. The multilevel approach allowed for the assessment of both individual and contextual factors on malaria infection, recognizing that outcomes may be shaped by variables at multiple levels, including individual characteristics and community-level determinants.

The community-based approach ensured that the findings represented a diverse range of outlooks within the broader population, making them highly relevant for public health initiatives focused on enhancing education and healthcare access for children under five. The study employed a cross-sectional design that facilitated data collection at a single point in time, enabling the identification of malaria infections and the examination of associations between variables without implying causation. Data collected from 2016 to 2022 were analyzed to strengthen reliability and provide a comprehensive understanding of trends and patterns related to malaria infection in this age group, using a multilevel mixed-effects approach to account for the hierarchical structure of the data.

### Population and eligibility criteria

The source population comprised children under 5 years of age in 17 SSA countries, while the study population included all children tested for malaria who had received their test results within the designated enumeration areas analyzed in the study. Eligible participants were those who had been tested and received their results during healthcare visits, ensuring that the data accurately captured the experiences of healthcare providers and mothers engaged in child healthcare.

### Data source and sampling procedure

The data for this study were obtained from the MIS conducted in 17 SSA countries. As such, the author’s team conducted a secondary data analysis. The MIS, supported by The DHS Program, provides extensive datasets covering various health indicators, including mortality, morbidity, child health, and malaria-related complications in children. These surveys are conducted approximately every 5 years. Each country’s survey consists of distinct datasets, which were combined to examine malaria infection and its determinants among children under five who had been tested for malaria infection [26].

The MIS employs a stratified two-stage cluster sampling design. In the first stage, enumeration areas are selected, and in the second stage, a sample of households is generated from each enumeration area. For detailed information about the MIS methodology, including survey instruments, sample design, and data tabulation plans, please refer to the MIS methodology overview available on The DHS program website.

For this study, the person record (PR) dataset was utilized to extract both dependent and independent variables relevant to the analysis. The variable ‘child malaria infection’ (hml35) from the PR dataset was recoded to create the outcome variable representing malaria infection in children under five. A binary logistic regression model was applied to analyze the data and identify factors associated with malaria infection in children under five. Determinants were reported as adjusted odds ratios with a 95% confidence level. The variable selection process for the multivariable analysis was based on a *p*-value threshold of 0.25 in the univariate analysis. This ensured the inclusion of potentially relevant variables for a thorough assessment of their relationship with malaria infection among children under five. In the univariate analysis, all variables with a *p*-value < 0.25 were considered candidates for inclusion in the multivariable analysis. In the subsequent multivariable logistic regression, variables with *p*-values < 0.05 were deemed statistically significant. The final analysis incorporated a total weighted sample of 51,457 children under five, ensuring the results accurately represent the population and reflect the complexities of malaria infection. The samples were weighted by specific demographic and population characteristics to ensure they accurately represent the broader population in each country surveyed. This process typically considers factors such as age, sex, and geographic distribution in the population, along with adjustments for non-response and sample design (Table 1).

**Table 1.** Sample size for malaria prevalence rates among under five children in all study countries of SSA, MIS 2016–2022.

Country	Year of survey	Weighted sample (n)	Weighted sample (%)
Burkina Faso	2017	2,261	4.39%
Cameroon	2022	2,558	4.97%
Ghana	2019	1,584	3.08%
Guinea	2021	2,058	4.21%
Kenya	2022	8,070	15.48%
Liberia	2022	1,521	2.96%
Madagascar	2016	3,935	7.65%
Mali	2017	3,283	6.38%
Malawi	2021	1,286	2.50%
Mozambique	2018	2,156	4.19%
Nigeria	2021	6,162	11.98%
Niger	2021	2,096	4.07%
Sierra Leone	2016	4,254	8.27%
Senegal	2021	633	1.23%
Togo	2017	1,869	3.63%
Tanzania	2017	3,178	6.18%
Uganda	2018	4,553	8.85%
Weighted sample size		51,457	100%

### Study variables

The primary outcome was the prevalence of malaria infection among under five children who had tested for malaria infection. This malaria infection on under five children was assessed by recoding the variable (hml35) from the person record dataset.

At the individual level, variables included child age in years, gender of children, maternal education, number of under five children in house, has a bed net, use for child bed net for sleep, sprayed against mosquito in last 12 months, household wealth index (The ‘Household Wealth Index’ was obtained from the dataset based on income information provided in the MIS or DHS surveys, which typically assess various economic indicators to categorize household wealth groups).

The ‘Household Media Exposure’ variable was constructed by summing the frequency of exposure to newspapers, radio, and television. The survey data included key housing characteristics categorized as improved and unimproved: the ‘main roof material’ (improved: metal or tiles; unimproved: thatch), the ‘main wall material’ (improved: bricks or concrete; unimproved: mud), and the ‘source of drinking water’ (improved: piped water, boreholes, or protected wells; unimproved: unprotected wells or surface water) [27]. These factors help assess living conditions and their potential impact on health outcomes. Community variables are defined as factors that influence malaria infection at the community level, including residence (classified as urban or rural), community media exposure (categorized as low or high engagement with health information, through radio, newspaper and television), community illiteracy (measured by the percentage of the population unable to read or write), community poverty (determined by poverty indicators classified as low poverty when indicators suggest favorable economic conditions and as high poverty when multiple indicators indicate financial struggle) [28], and geographic level above sea level (reflecting altitude’s impact on mosquito breeding patterns) [29]. The process to create these variables involves importing the dataset into STATA, generating the necessary binary or categorical variables based on established thresholds, and exporting the modified dataset for further analysis. These comprehensive variables were essential for analyzing the factors associated with malarial infection among under five children in the selected 17 SSA countries.

### Data processing and statistical analysis

The data were sourced from the latest MIS datasets and cleaned, re-coded, and analyzed using statistical software. Prior to performing any statistical analysis, sampling weights, primary sampling units, and strata were applied to ensure the survey’s representativeness and account for its sampling design when calculating standard errors, thereby producing reliable statistical estimates. The weighting variable (hml35) served as a normalized relative weight tailored to the specific survey.

For pooled data, the individual standard weight for malaria infection was de-normalized by adjusting it according to each country’s sampling fraction, calculated as follows: (Under five malarial infected children adjusted weight = *hml35 × (total malarial infected children age from 1 month to 5 years in the country at the time of the survey)/(number of malarial infection under five children))*. Given the hierarchical structure of MIS data, standard logistic regression assumptions – such as independence of observations and homogeneity of variance – were not met. Children grouped within clusters may share similar characteristics, influencing these assumptions. To account for between-cluster variability in assessing the association with malarial infection, a multilevel mixed-effects logistic regression model was implemented.

The multilevel mixed-effects logistic regression analysis incorporated four models: the null model (including only the outcome variable), Model I (accounting for individual-level variables), Model II (considering community-level variables), and Model III (integrating both individual- and community-level variables). The null model was utilized to examine the variability in malaria infection prevalence across clusters. Model I explored the relationship between individual-level variables and the outcome, while Model II assessed the influence of community-level variables. In the final model, Model III, both individual and community-level variables were simultaneously included with the outcome variable. Model fitness was evaluated, and the model with the lowest deviance and highest log-likelihood ratio was identified as the best-fit model.

### Random effects (measures of variation)

Random effects, or measures of variation for the outcome variables, were estimated using intra-class correlation coefficient (ICC), the median odds ratio (MOR) and proportional change in variance (PCV). The ICC and PCV were computed to assess the variation between clusters. By treating clusters as random variables, the ICC quantifies the variation in malaria prevalence rates across clusters, calculated as follows; ICC = $\frac{\mathrm{VC}}{\mathrm{VC}+3.29}\times 100$% [30]. The Median Odds Ratio (MOR) is the median value of the odds ratio which quantifies the variation or heterogeneity in malaria prevalence rates between clusters in terms of odds ratio scale and is defined as the median value of the odds ratio between the cluster at high likelihood of malaria Under five prevalence rates and cluster at lower risk when randomly picking out individuals from two clusters; MOR = e ^0.95√VC^ Moreover, the PCV demonstrates the variation in the malaria prevalence rates explained by determinants and computed as;

PCV = $\frac{\mathrm{Vnull}-\mathrm{Vc}}{\mathrm{Vnull}}\times 100$%; where V-null is the variance of the null model and VC is the cluster-level variance. Fixed effects were utilized to estimate the association between the likelihood of malaria and both individual and community-level independent variables [31,32].

The strength of these associations was presented using adjusted odds ratios (AOR) and 95% confidence intervals, with a significance threshold set at *p* < 0.05. Due to the nested nature of the model, the deviance statistic (−2 log likelihood ratio) was employed to compare models, selecting the one with the lowest deviance as the best fit. Additionally, the variables included in the models were checked for multi-collinearity by measuring the variance inflation factors (VIF), with results falling within acceptable limits of 1–10, ensuring the robustness of the analysis [33].

## Result

### Sociodemographic and economic characteristics of study participants

A total of 51,457 children were included in the analysis. More than three-quarters of the participants were aged between 2 and 5 years, and approximately half of the children were female. Notably, over half of the mothers had no formal education. In terms of household conditions, three-quarters of the families possessed bed nets; however, around one-third of these households did not utilize them for their children. Regarding economic conditions, nearly half of the households were classified as having a poor wealth index. Additionally, about 74.3% of the participants resided in rural areas across 17 sub-Saharan African countries (see Table 2).

**Table 2.** Socio-demographic and economic characteristics of study participants.

Individual level variables	Category	Frequency (n)	Percent (%)
Child Age In years	<6 month	383	0.74%
	6 month-1 year	3,524	6.85%
	1–2 years	7,491	14.56%
	>2-5 years	40,059	77.85%
Gender of children	female	25,773	50.09%
	male	25,684	49.91%
Maternal education	No formal education	29,923	58.15%
	Primary	12,222	23.75%
	Secondary and above	9,312	18.10%
Number of under 5 years children in house	1	13,891	27.16%
	2–3	28,272	54.94%
	>3	9,294	18.06%
Has a bed net	No	11,160	21.69%
	Yes	40,297	78.31%
Use for Child bed net for sleep	No	16,907	32.86%
	Yes	34,550	67.14%
Sprayed against mosquito in last 12 months	No	49,634	96.46%
	Yes	1,823	3.54%
House hold wealth index	Poor	25,283	49.13%
	Middle	10,447	20.30%
	Rich	15,727	30.56%
House hold media exposure	No	21,934	42.63%
	Yes	29,519	57.37%
Main floor material	Unimproved	29,101	56.55%
	Improved	22,356	43.45%
Main wall material	Unimproved	27,115	52.69%
	Improved	24,342	47.31%
Main roof material	Unimproved	27,115	52.69%
	Improved	24,342	47.31%
Source of drinking water	Unimproved	21,914	42.59%
	Improved	29,543	57.41%
**Community level factors**			
Household Residence	Urban	13,226	25.70%
	Rural	38,231	74.30%
Community level of Illiteracy	Low	6,522	12.67%
	High	44,935	87.33%
Community level of poverty	Low	8,478	16.48%
	High	42,979	83.52%
Community level of media exposure	Low	12,467	24.23%
	High	38,990	75.77%
Country category above sea level	Lowland	18,368	35.70%
	Sub-humid	19,798	38.47%
	Highland	13,291	25.83%

### Prevalence of under-five years’ children malaria infection in SSA

**Figure 3.**
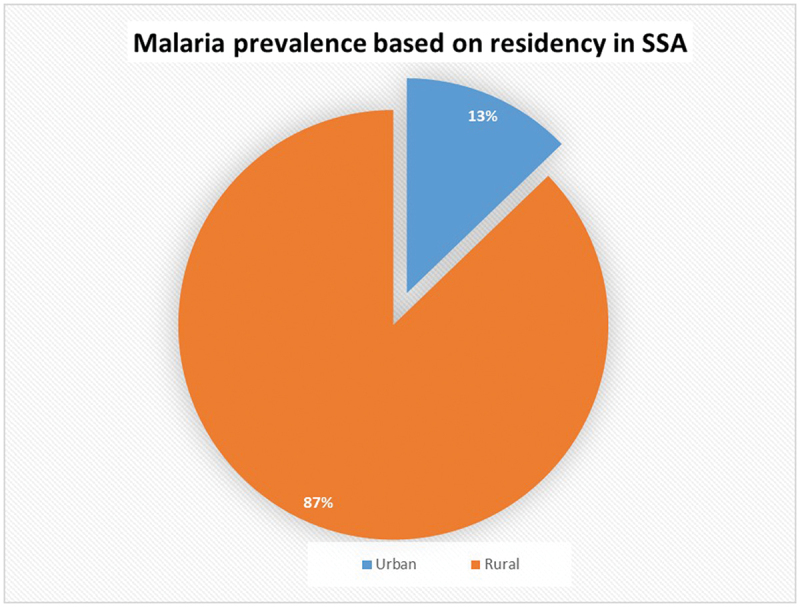
Malaria prevalence in children under 5 years of age in 17 SSA countries, stratified by residency in either a local or urban area.

The overall prevalence of malaria infection among children under five across the 17 study countries was 36.51% (95% CI: 36.09–36.93). A greater burden was observed in rural areas, with 38,231 cases, compared to 13,226 cases in urban areas (Figure 3).

### Variation in malaria prevalence among children under 5 years of age in 17 SSA countries

**Figure 4.**
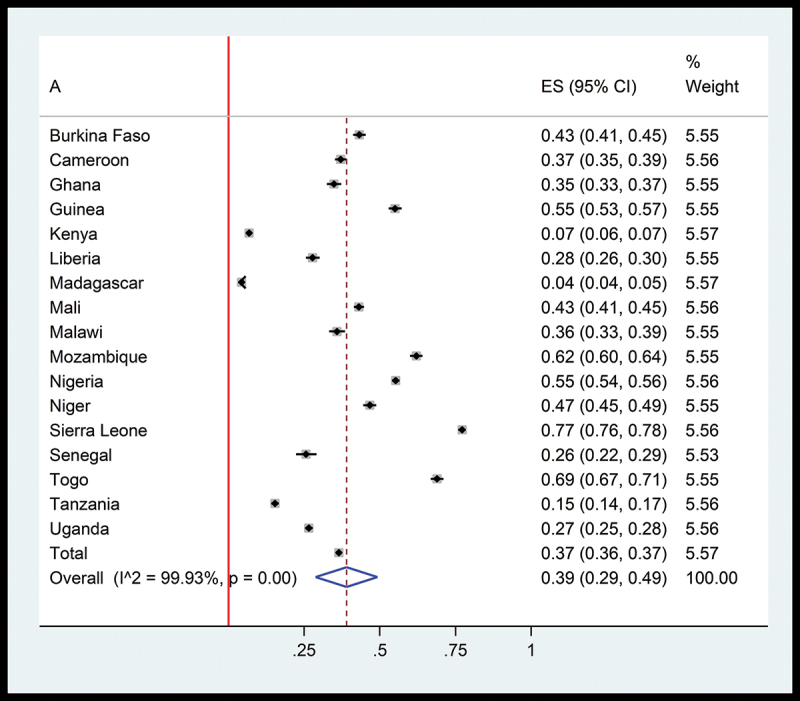
Malaria prevalence among children under 5 years of age in all 17 study countries of SSA.

Malaria prevalence among children under five in 17 SSA countries from 2016 to 2022 shows significant variation across countries. Sierra Leone exhibits the highest prevalence at 77%, while Madagascar demonstrates the lowest at 4%. The high I^2^ statistic of 99.93% indicates substantial heterogeneity among studies, reflecting varied socio-economic and health system factors influencing malaria transmission (Figure 4).

### Random effect (measures of variation) and model fitness

Findings from the null model indicated significant differences in malaria prevalence among communities, with a variance of 2.938897. In this model, approximately 47.2% of the total variation in malaria prevalence occurred at the cluster level, attributable to community-level factors. The null model also exhibited the highest median odds ratio (MOR) of 5.1, suggesting that individuals in communities with higher malaria prevalence had 5.1 times greater odds of being infected compared to those in communities with lower prevalence. The intraclass correlation coefficient for Model I showed that 49.4% of the variation in malaria prevalence was attributable to disparities between communities. As we incorporated community-level variables to create Model II, the ICC indicated that cluster variations accounted for 20.91% of the differences in malaria prevalence. In the final model (Model III), approximately 72.65% of the variation in malaria prevalence was attributed to both individual and community-level factors. Model fitness was evaluated using log-likelihood ratio and deviance, with Model III emerging as the best-fitting model due to its lowest deviance (51,803.366) and highest log-likelihood (−25,901.683) (Table. 3).

**Table 3.** Model comparison and random effect analysis for malaria prevalence rates among under five children in all study countries of SSA, MIS 2016–2022.

Parameter	Null model	Model I	Model II	Model III
variance	2.938897	2.218538	0.8696485	0.8038238
ICC	47.2%	49.4%	20.91%	19.63%
MOR	5.1	4.12	2.43	2.34
PCV	Reference	24.5%	70.43%	72.65%
**Model fitness**				
LLR	−30,667.298	−28,497.183	−26,831.629	−25,901.683
Deviance	61,334.596	56,994.366	53,663.258	51,803.366

ICC: intra-cluster correlation, LLR: log-likelihood ratio, MOR: median odds ratio, PCV: proportional change in variance.

### Logistic regression analysis of individual-level and community level factors associated with malaria prevalence rates among under five children

The results showed that, for child age, those under 6 months had an AOR of 0.26 (0.19, 0.36), while children aged 6 months-1 year had an AOR of 1.31 (1.21, 1.41) and those aged 1–2 years had an AOR of 1.51 (1.43, 1.60); children over 2–5 years served as the reference group with an AOR of 1.00. In terms of gender, females exhibited an AOR of 0.84 (0.81, 0.88) compared to males at 1.00. Regarding maternal education, those with no formal education had an AOR of 1.00, while primary education correlated with an AOR of 0.65 (0.62, 0.68) and secondary education and above showed an AOR of 0.58 (0.54, 0.62). Additional factors indicated in (Table 4), include socioeconomic status and access to healthcare, both of which significantly influenced the outcomes.

**Table 4.** Multivariable multilevel logistic regression analysis of individual-level and community-level factors associated with malaria prevalence rates among under five children in all study countries of SSA, MIS 2016–2022.

Individual-level variables		Model I (AOR = 95%)	Model II (AOR = 95%)	Model III (AOR = 95%)
Child Age In years	<6 month	0.26(0.19,0.36)		0.28(0.20,0.38)*
	6 month-1 year	1.31(1.21,1.41)		1.19(1.09,1.29)*
	1–2 years	1.51(1.43,160)		1.46(1.37,1.54)*
	>2-5 years	1		
Gender of children	Female	0.84(0.81,0.88)		0.83(0.79,0.87)
	Male	1		
Maternal education	No formal education	1		
	Primary	0.65(0.62,0.68)		0.84(0.79,0.89)*
	Secondary and above	0.58(0.54,0.62)		0.61(0.57,0.65)*
Number of another under 5 years children in house	1	1		
	2–3	1.39(1.33,1.46)		1.23(1.17,1.29)*
	>3	1.67(1.56,1.79)		1.41(1.32,1.52)*
Has a bed net	No	1		
	Yes	0.91(0.85,0.97)		0.94 (0.87,1.02)
Use for Child bed net for sleep	No	1		
	Yes	1.11(1.05,1.18)		0.80(0.76,0.84)*
Sprayed against mosquito in last 12 months	No	1		
	Yes	0.34(0.29,0.38)		0.34(0.29,0.39)*
House hold wealth index	Poor	3.71(3.45,3.98)		1.94(1.80,2.10)*
	Middle	2.46(2.31,2.63)		1.57(1.46,1.69)*
	Rich	1		
House hold media exposure	No	1		
	Yes	0.91(0.86,0.95)		0.84(0.79,0.89)*
Main floor material	Unimproved	1		
	Improved	1.20(1.14,1.27)		0.98(0.93,1.04)
Main wall material	Unimproved	1		
	Improved	1.09(1.03,1.14)		0.95(0.89,1.01)
Source of drinking water	Unimproved	1		
	Improved	0.97(0.93,1.01)		0.96(0.92,1.01)
**Community level factors**				
Household Residence	Urban	1		
	Rural		4.31(4.06,4.58)	2.69(2.51,2.88)*
Community level of Illiteracy	Low	1		
	High		1.79(1.44,2.21)	1.76(1.43,2.17)*
Community level of poverty	Low	1		
	High		1.82(1.51,2.19)	1.22(1.02,1.47)*
Community level of media exposure	Low	1		
	High		1.16(0.95,1.41)	1.13(0.93,1.37)
Country category based on sea level	Lowland		14.9(13.7,16.3)	14.7(13.4,16.1)*
	Sub-humid		5.53(5.07,6.03)	5.63(5.12,6.16)*
	Highland	1		

Written in bold and asterisks (*) indicates the significant factors associated with outcome variables.

## Discussion

The malaria infection prevalence across the 17 SSA countries analyzed stands at **36.5%**, underscoring a substantial public health burden that requires sustained care and strengthened prevention efforts. This study examined the factors associated with malaria infection among children under five using recent MIS data, revealing significant associations between individual-level factors (child age, maternal education, household size, bed net use, mosquito spraying, wealth index, media exposure, and housing materials) and community-level factors (residence, illiteracy, poverty, media exposure, and geographic location). Results indicate that children aged 6 months to 2 years are at higher risk of malaria infection compared to those aged 2–5 years (Model II). This finding is consistent with existing literature, which suggests that infants under 6 months may benefit from maternal antibodies, while older children gradually acquire immunity through repeated exposure. The elevated vulnerability of children in the 6–24-month age group highlights a critical window where protection is limited and immunity is not yet established. Although the sample size for this subgroup was relatively small, which may limit interpretability, the implications remain significant: without targeted interventions, these children face a disproportionate risk of severe outcomes. The convergence of individual and community-level determinants demonstrates that malaria control strategies must urgently integrate household-level practices with broader structural and contextual interventions to reduce the burden among under-fives [2].

The protective effect observed in children under 6 months (AOR = 0.28) supports the understanding that being under 6 months significantly reduces the likelihood of malaria infection by 72% compared to children older than 2 years highlights the protective impact of early infancy [34]. Additionally, increased attention and care during the first 6 months, such as exclusive breastfeeding, likely contribute to this protection, as supported by other studies [35,36]. Further exploration into the specific mechanisms of maternal immunity and the role of nurture in early infancy could provide deeper insights into malaria prevention strategies for young children [37]. This effect may be attributed to malarial infection factors, including maternal immunity passed through breast milk and antibodies [38].

Maternal education emerged as a significant protective factor against malaria infection. Children whose mothers had primary or secondary education were less likely to be infected compared to those whose mothers had no formal education (Model II). This finding is supported by numerous studies demonstrating that educated mothers are more likely to adopt preventive measures, seek timely treatment, and possess greater health knowledge [39,40]. A study in the Democratic Republic of Congo found that higher maternal education was associated with lower malaria prevalence among children [41]. Similarly, a study across 11 SSA countries highlighted that children whose mothers had secondary education had a significantly lower risk of malaria infection [42].

Household size, specifically, the number of under-five children in the house, was positively associated with malaria infection. This could be due to increased exposure within the household and resource constraints [43]. In Model II, bed-net ownership did not show a significant protective effect after adjusting for community-level factors. While ownership indicates resource availability, the critical protective impact of bed-net use for child sleep was significant, with an AOR of 0.80. This finding underscores that, while bed nets can be effective in preventing malaria, their benefits depend on proper and consistent usage.

Therefore, creating awareness alongside improving access to bed nets is essential for effective malaria prevention. Educational campaigns must inform families about the correct use of bed nets and their benefits, particularly for young children who are especially vulnerable to malaria. By combining increased access to bed nets with targeted awareness programs, communities are more likely to adopt protective behaviors, significantly reducing malaria risk and improving health outcomes for children and families [44,45]. Furthermore, household spraying against mosquitoes in the last 12 months was strongly protective (AOR = 0.34), underscoring the effectiveness of indoor residual spraying [18,46].

Household wealth index was a significant determinant of malaria infection. Children from poor households had higher odds of malaria infection compared to those from rich households (Model II). This finding is consistent with a systematic review and meta-analysis that found low wealth to be associated with increased malaria risk [47,48]. Poorer households often have limited access to healthcare, preventive measures, and better housing [49]. Similarly, household media exposure was protective (AOR = 0.84), indicating that access to information through mass media plays a crucial role in malaria prevention [50,51].

Although unimproved floor and wall materials were expected to elevate malaria risk, the fully adjusted model (Model II) revealed no significant association. This may reflect the influence of other factors or limitations in how housing quality was measured in the MIS data. Nonetheless, prior research consistently shows that improved housing conditions are linked to lower malaria risk. Therefore, the findings of this study do not contradict earlier evidence but rather highlight that housing effects may be context-specific or masked by other determinants [52].

Spraying of homes reduced malaria infections by 64% and this supported by other study [53]. The source of drinking water did not show a significant association with malaria infection in our analysis. However, other studies have found that unimproved water sources and sanitation conditions are associated with increased malaria risk [54]. This discrepancy might be due to differences in study settings or the specific variables used.

Consistent with existing literature, our study found that children residing in rural areas had significantly higher odds of malaria infection compared to those in urban areas (AOR = 2.69). This is likely due to factors such as greater mosquito density, poorer access to healthcare, and lower socioeconomic status in rural settings [13,55].

Higher community levels of illiteracy and poverty were associated with increased odds of malaria infection (Model II). These findings underscore the importance of addressing socioeconomic disparities at the community level to reduce malaria transmission [56].

The country category based on sea level was a strong predictor of malaria infection. Children living in lowland and sub-humid areas had significantly higher odds of malaria compared to those in highland areas. This is consistent with the understanding that malaria transmission is influenced by climatic and environmental factors, with lowland areas often providing more suitable breeding grounds for mosquitoes [57,58].

## Strengths and limitations

This study leverages large, nationally representative data from 17 SSA countries and applies robust multilevel mixed-effects models, enabling a comprehensive analysis of both individual and community determinants of malaria infection. Limitations include reliance on tested children only, possible underestimation of prevalence. Despite these, the breadth and methodological rigor provide strong evidence to guide targeted interventions and policy action.

## Conclusion

This study provides valuable insights into the complex interplay of individual and community-level factors on malaria prevalence among children under 5 years of age in SSA. Our findings emphasize the need to address socioeconomic disparities, improve access to healthcare and preventive measures, and implement targeted interventions at both individual and community levels. Strong policy implications arise from these findings: governments and health organizations must prioritize investments in healthcare infrastructure, especially in high-prevalence areas, to ensure equitable access to malaria prevention and treatment.

Integrating community education programs focused on mosquito control, the importance of testing and treatment, and the correct use of preventive tools is essential for empowering families to take proactive measures. Specifically, enhancing access to bed nets and insecticides, while providing training on their proper utilization, can significantly benefit children under five. Furthermore, promoting awareness about the critical role of regular bed net use and the correct application of insecticide sprays can heighten protection against malaria. Policies aimed at reducing poverty and improving household wealth could also make a substantial difference in malaria outcomes, ultimately leading to healthier communities and reduced mortality rates in vulnerable populations.

## Data Availability

Third party data was obtained for this study from The DHS Program (https://dhsprogram.com/). Data may be requested from The DHS Program after creating an account and submitting a concept note. More access information can be found on The DHS Program website (https://dhsprogram.com/data/Access-Instructions.cfm). The data set is openly available upon permission from the MEASURE DHS website (https://www.dhsprogram.com/data/available-datasets.cfm). Interested researchers can access these data in the same manner as the authors, and no special access privileges were granted that would not be available to others.
